# Quantitative chemical exchange saturation transfer (CEST) MRI of glioma using Image Downsampling Expedited Adaptive Least-squares (IDEAL) fitting

**DOI:** 10.1038/s41598-017-00167-y

**Published:** 2017-03-07

**Authors:** Iris Yuwen Zhou, Enfeng Wang, Jerry S. Cheung, Xiaoan Zhang, Giulia Fulci, Phillip Zhe Sun

**Affiliations:** 1 Athinoula A. Martinos Center for Biomedical Imaging, Department of Radiology, Massachusetts General Hospital and Harvard Medical School, Charlestown, MA USA; 2 Department of Radiology, 3rd Affiliated Hospital, Zhengzhou University, Henan, China; 3 Molecular Neuro-oncology Laboratories, Department of Neurosurgery, Massachusetts General Hospital and Harvard Medical School, Boston, MA 02124 USA

## Abstract

Chemical Exchange Saturation Transfer (CEST) MRI is sensitive to dilute metabolites with exchangeable protons, allowing tissue characterization in diseases such as acute stroke and tumor. CEST quantification using multi-pool Lorentzian fitting is challenging due to its strong dependence on image signal-to-noise ratio (SNR), initial values and boundaries. Herein we proposed an Image Downsampling Expedited Adaptive Least-squares (IDEAL) fitting algorithm that quantifies CEST images based on initial values from multi-pool Lorentzian fitting of iteratively less downsampled images until the original resolution. The IDEAL fitting in phantom data with superimposed noise provided smaller coefficient of variation and higher contrast-to-noise ratio at a faster fitting speed compared to conventional fitting. We further applied the IDEAL fitting to quantify CEST MRI in rat gliomas and confirmed its advantage for *in vivo* CEST quantification. In addition to significant changes in amide proton transfer and semisolid macromolecular magnetization transfer effects, the IDEAL fitting revealed pronounced negative contrasts of tumors in the fitted CEST maps at 2 ppm and −1.6 ppm, likely arising from changes in creatine level and nuclear overhauser effects, which were not found using conventional method. It is anticipated that the proposed method can be generalized to quantify MRI data where SNR is suboptimal.

## Introduction

Chemical Exchange Saturation Transfer (CEST) MRI is a sensitive imaging technique for detecting compounds containing exchangeable protons^1–3^. Such labile protons can be selectively saturated by a radiofrequency (RF) pulse and subsequently transfer the saturation to the bulk water signal via proton chemical exchange, resulting in substantial sensitivity enhancement. CEST imaging has been demonstrated in mapping low-concentration metabolites such as creatine^4^, glucose^5, 6^, glutamate^7^ and changes in microenvironment properties such as temperature^8^ and pH^9–11^, promising in a host of *in vivo* applications such as imaging of ischemic stroke^12–15^ and tumor^16–21^.

Quantitative CEST MRI is typically performed by analyzing the Z-spectrum acquired through sweeping RF saturation around the bulk water resonance, offset by offset. *In vivo* CEST quantification remains challenging due to concomitant effects such as RF spillover (direct water saturation), semisolid macromolecular magnetization transfer (MT) and nuclear overhauser effects (NOE). As such, the conventional asymmetry analysis (MTR_asym_), which has been widely used to suppress spillover and MT effects, measures a mixed contribution. Hence, resolving individual contributions is necessary. To avoid asymmetric MT effect, a three-offset approach that subtracts the label image from the average of two boundary images that have an equal offset shift from the label image was proposed to quantify amide proton transfer (APT) and NOE effects^22, 23^. However, the linear assumption underlying the three offset method is oversimplified, which likely underestimates the APT and NOE effects. Recently, least-squares Z-spectral fitting approaches have been employed to resolve multi-pool contributions, including APT, spillover, MT and NOE^18, 24, 25^ or enzyme-responsive CEST agent^26^. For example, Heo *et al.* fitted wide-offset data with a super-Lorentzian lineshape to estimate the semisolid MT signal and quantified APT and NOE effects by subtracting the fitted MT signal from the Z-spectrum^27, 28^. Analytical studies confirmed that individual CEST effect, including the MT effect within a small range of frequency offsets, can be approximated by a Lorentzian line shape in a Z-spectrum^29–31^. Therefore, an alternative approach is to fit the Z-spectra at low irradiation powers using a multi-pool Lorentzian model^29, 32, 33^. The reliability of these approaches depends strongly on the signal-to-noise ratio (SNR), which may be compromised by spatiotemporal resolution. Moreover, conventional least-squares fitting is sensitive to the initial values and boundary values, which can lead to inaccurate fitting results, if not properly selected.

**Figure 1:**
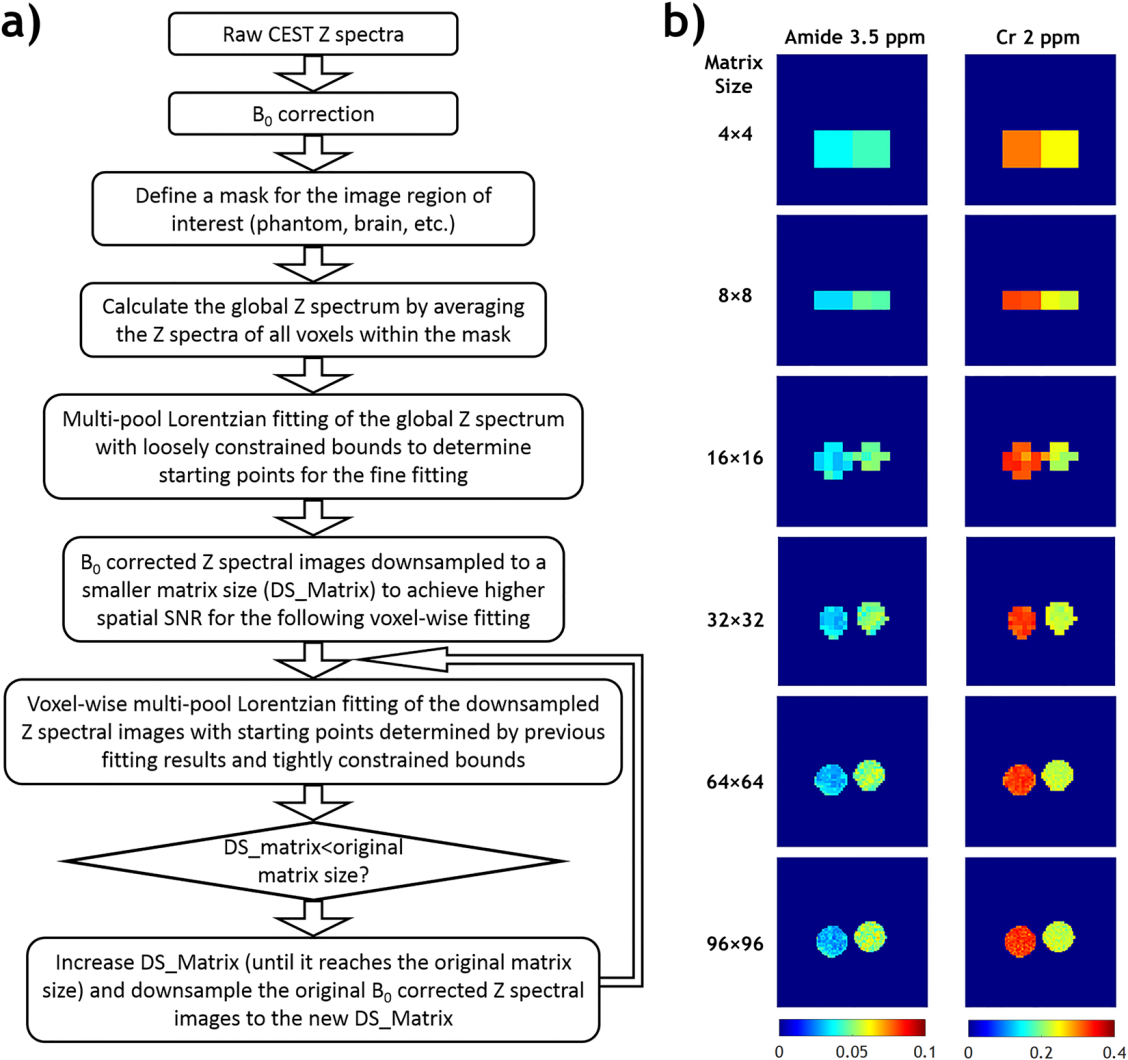
(**a**) Flow chart of data processing steps of the Image Downsampling Expedited Adaptive Least-squares (IDEAL) fitting approach. (**b**) The intermediate fitting results of iteratively less downsampled images from a two-compartment CEST phantom.

In this study, we proposed an Image Downsampling Expedited Adaptive Least-squares (IDEAL) fitting algorithm to improve the reliability of *in vivo* CEST MRI quantification. Figure 1a shows the flow chart of the proposed IDEAL fitting. Specifically, it uses globally averaged Z-spectrum for initial fitting, which are more reliable given the high SNR and relaxed boundary constraints. The initial fitting results are then used as initial values for subsequent fitting of substantially downsampled images. The resolution of downsampled images is increased iteratively and fitted with the fitting results from the previous downsampled images as renewed initial values until the desired image resolution is reached (Fig. 1b). We first evaluated the IDEAL fitting approach with conventional voxel-wise fitting in a CEST phantom superimposed with different levels of simulated noise. The fitting quality of both methods were assessed via coefficient of variation (COV, i.e., S.D./mean), contrast-to-noise ratio (CNR), goodness of fit and fitting speed. We then applied the IDEAL fitting *in vivo*, and quantified the complex CEST effects in a rat model of glioma, demonstrating the advantage of the proposed IDEAL fitting algorithm.

## Results

### Phantom Study

**Figure 2:**
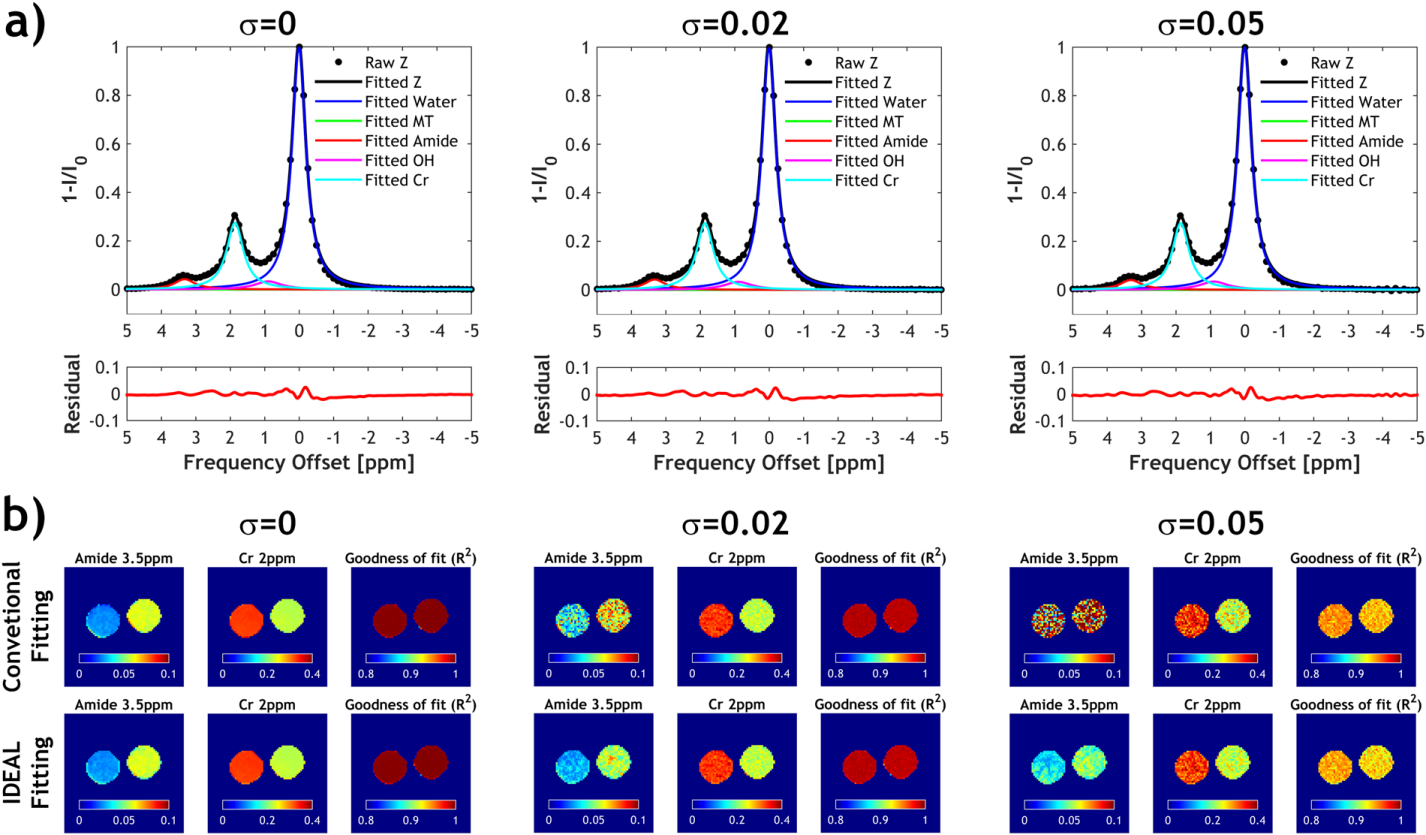
Conventional voxel-wise multi-pool Lorentzian fitting versus Image Downsampling Expedited Adaptive Least-squares (IDEAL) fitting in the two-compartment phantom (left: 50 mM Creatine (Cr) and 100 mM nicotinamide (amide); right: 100 mM Cr and 50 mM amide) with different levels of Gaussian noise added. (**a**) Multi-pool Lorentzian fit of the global Z spectra at different noise levels to determine initial values for IDEAL fitting. The global Z spectrum was obtained by averaging the Z spectra of all voxels within the phantom. (**b**) Fitted amide and Cr maps using conventional fitting or IDEAL fitting. The corresponding R^2^ maps were shown.

Figure 1b illustrates the intermediate fitting results of iteratively less downsampled images from a two-compartment CEST phantom using the proposed IDEAL fitting approach. Figure 2 compares the proposed IDEAL fitting with conventional fitting in the CEST phantom. The same initial values and boundary values were used in conventional fitting and in the fitting of global Z-spectrum (Fig. 2a) for determination of initial values in the IDEAL fitting approach. The global-Z spectrum was obtained by averaging the Z-spectra of all voxels within the phantom so it had substantially higher SNR than that of individual voxel. Consequently, the fitted CEST peaks of the global Z-spectrum were similar in amplitude, offset and linewidth without (σ = 0) or with added noise (σ = 0.02 and 0.05). Without added noise, the fitted amide and creatine (Cr) amplitude maps using conventional fitting and IDEAL fitting were comparable (Fig. 2b). Both methods showed high R^2^ maps. However, significant amount of noise appeared in the amplitude maps obtained from the conventional fitting after Gaussian noise superposition, indicating it's more susceptible to SNR degradation than the proposed IDEAL fitting algorithm.

**Figure 3:**
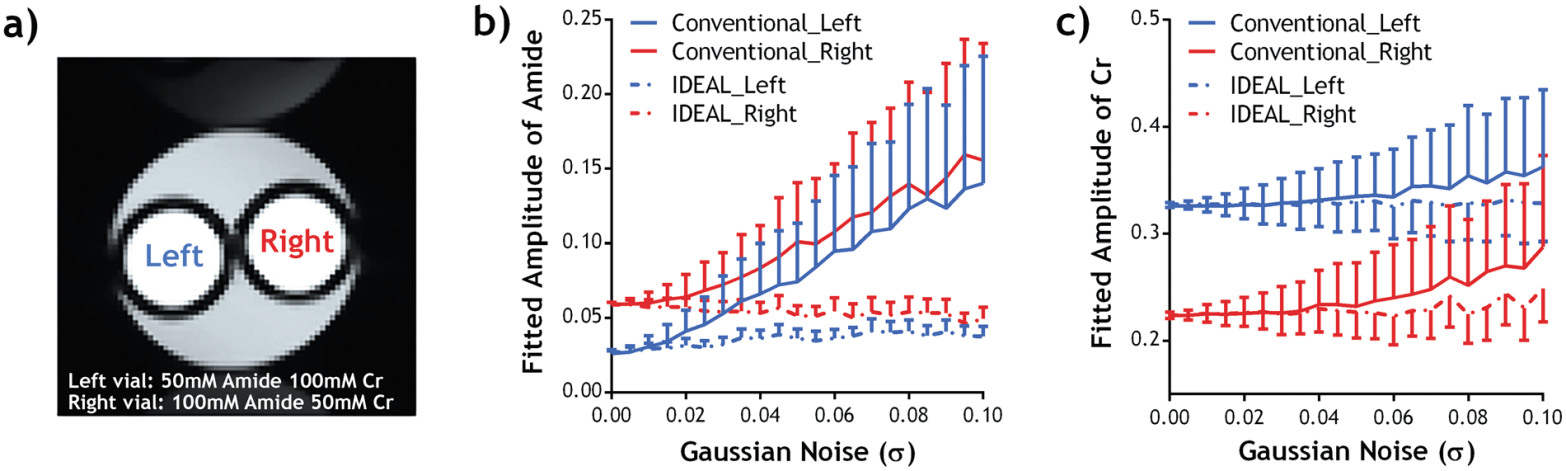
Comparison of fitted amplitudes from conventional fitting and IDEAL fitting under different noise level in (**a**) a phantom with two compartments containing different concentrations of amide and Cr. Fitted amplitudes of (**b**) amide and (**c**) Cr in each compartment was plotted as a function of standard deviation of added Gaussian noise (σ).

**Figure 4:**
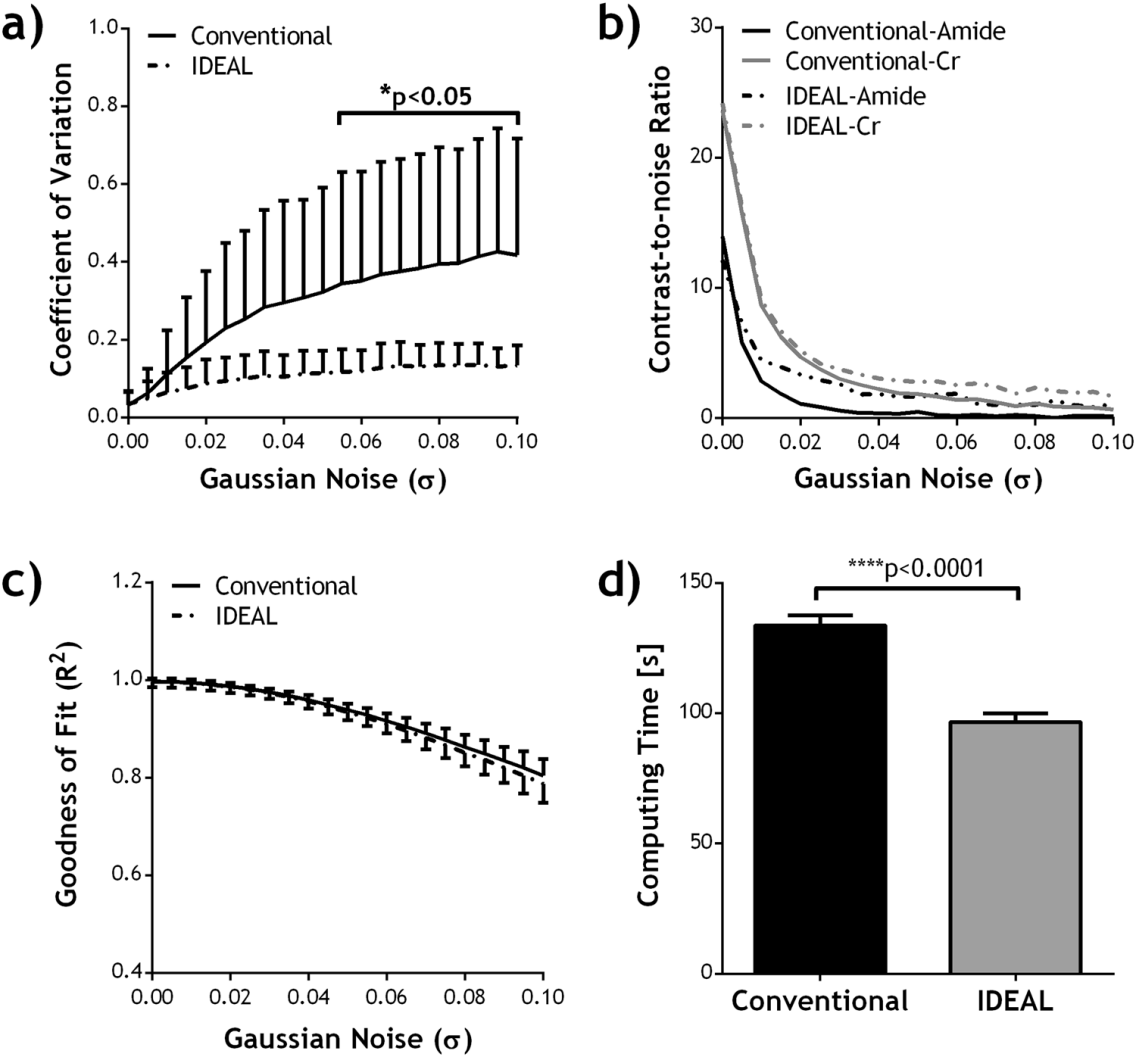
Comparison of fitting quality of conventional fitting and IDEAL fitting in the phantom, in terms of (**a**) coefficient of variation (COV, i.e., S.D./mean) in the fitted amplitude maps, (**b**) contrast-to-noise ratio (CNR) between the two compartments in the fitted amide and Cr amplitude maps, (**c**) R^2^ as a function of noise level (σ), and (**d**) computing time. Paired Student's t-test was performed between the two methods.

The performance of the IDEAL fitting was further evaluated under varied noise level (Figs 3 and 4). No significant difference was found in the fitted amplitudes of amide and Cr peaks between conventional fitting and IDEAL in the absence of simulated noise, and the results were used as the reference for further comparison. For the conventional fitting, the derived amplitudes increasingly deviated from the reference as the simulated noise level increased (Fig 3b,c). In contrast, the IDEAL fitting provided much more stable results, showing smaller deviation from the reference (P < 0.0001, paired t-test) with smaller SDs (P < 0.0001, paired t-test). For both fitting methods, the coefficient of variation (COV) in the fitted amplitude maps increased with the added noise level (Fig. 4a). However, the COV using IDEAL fitting was generally less affected by the noise and it was significantly smaller than that of conventional fitting for σ > 0.055. The contrast-to-noise ratio (CNR) between the two vials diminished with increased noise (Fig. 4b), but the CNR from IDEAL fitting was significantly higher than that of conventional fitting (P = 0.029, paired t-test). The goodness of fit (R^2^) of the two fitting methods was not significantly different but both gradually decreased with increased noise level (Fig. 4c). Importantly, the total computing time using IDEAL fitting was substantially smaller than that of conventional fitting at different noise level (P < 0.0001, paired t-test).

### Animal Study

**Figure 5:**
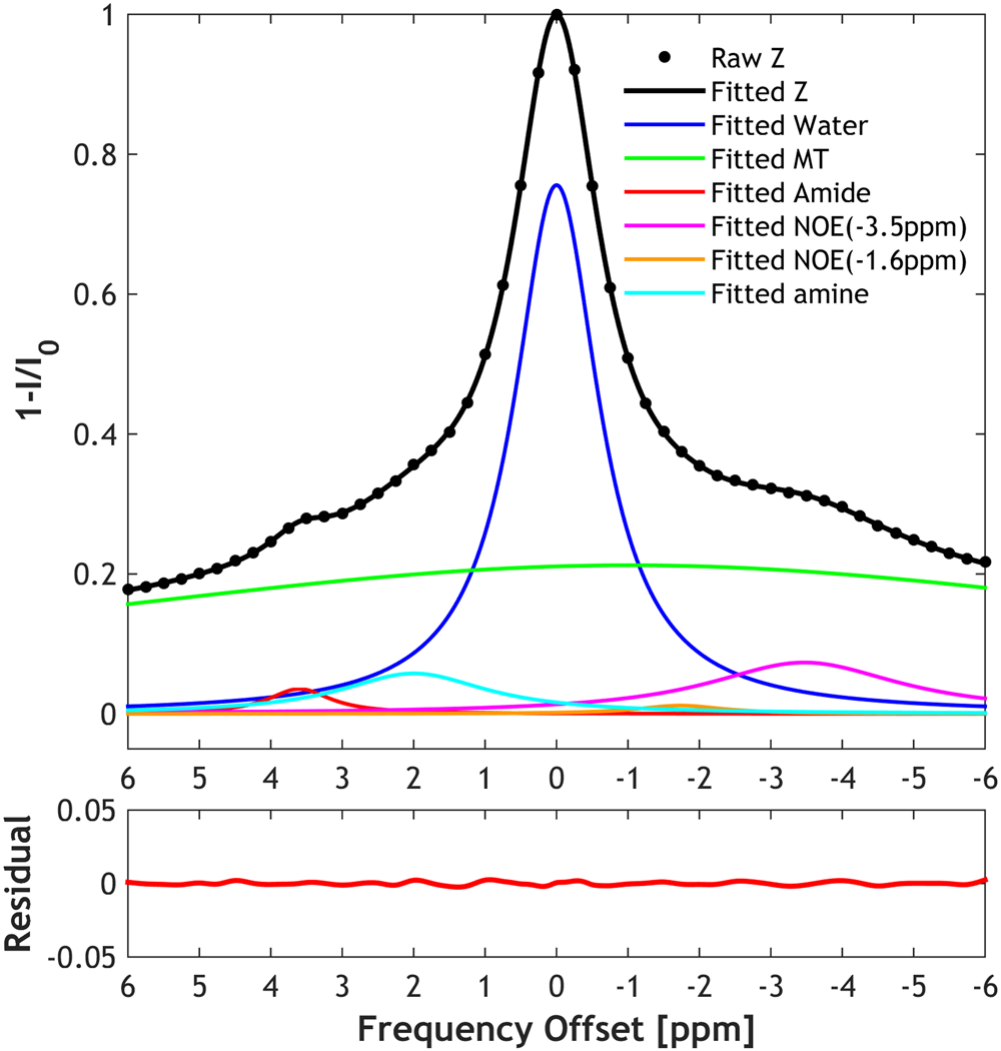
Multi-pool Lorentzian fit of the global Z spectra from a rat brain to determine initial values for IDEAL fitting, including saturation transfer effects from −3.5, −1.6, 2, 3.5 ppm as well as direct saturation and MT contributions.

**Figure 6:**
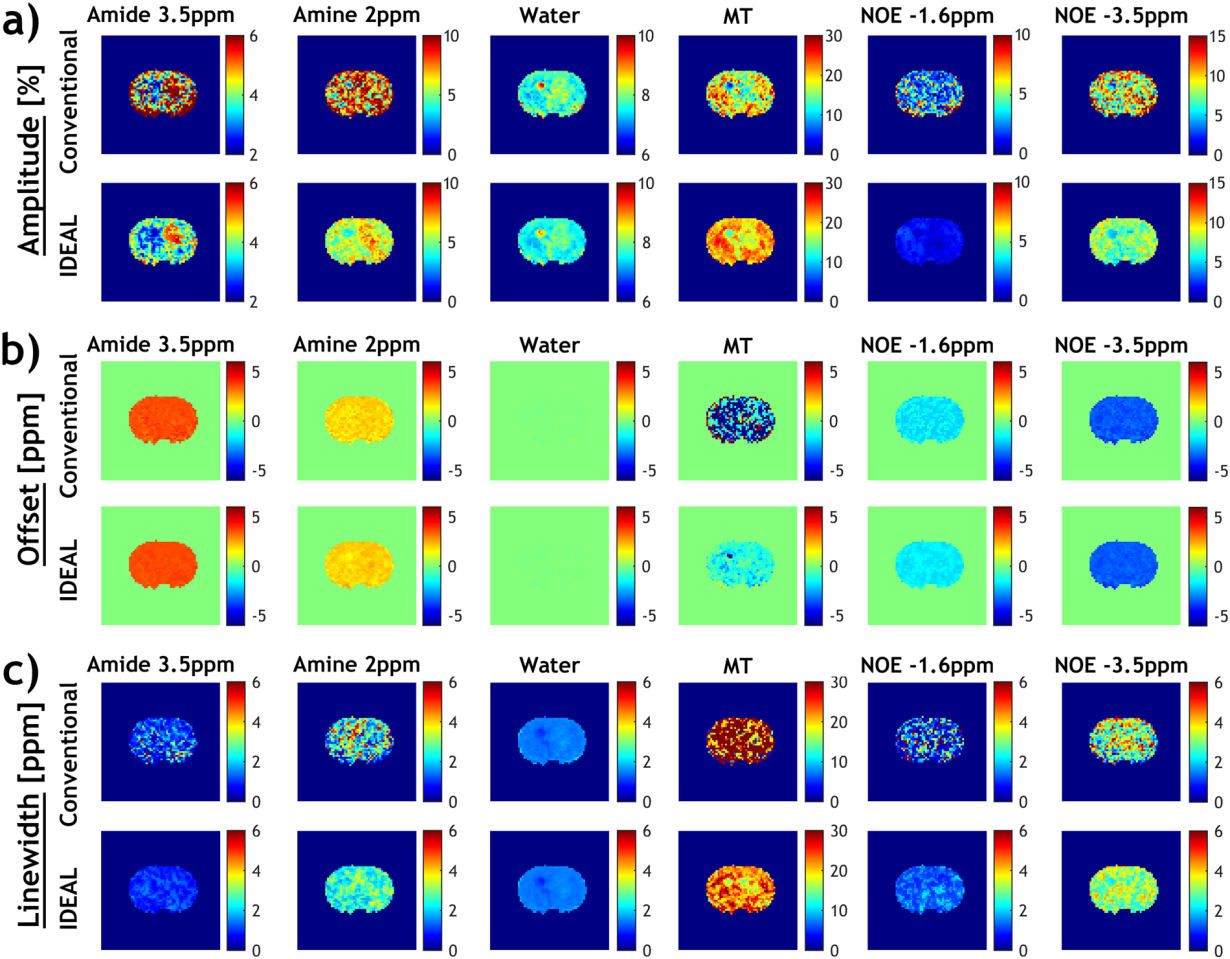
Fitted amplitude, frequency offset and linewidth maps of each pool using conventional fitting and IDEAL fitting in a representative rat brain with glioma.

We further applied the IDEAL fitting to quantify CEST MRI in a rat glioma model. Figure 5 shows the global Z-spectrum averaged from all voxels within the brain and it was fitted using a six-pool model. The fitting residual had no apparent peaks and was less than 0.26% over the frequency offset range. The fitting results were then used as the initial values for the iterative IDEAL fitting. Figure 6 shows the fitted amplitude, frequency offset and linewidth maps of each pool using both the conventional fitting and IDEAL fitting in a representative rat brain with glioma. The tumor appeared hyperintense in the fitted amide amplitude map using the IDEAL fitting (Fig. 6a) yet such contrast was less apparent and clear in the conventional fitting. Furthermore, the amine map using IDEAL fitting displayed a positive contrast which was not observed in the result from conventional fitting. For both fitting methods, water map showed relatively hyperintensity while MT maps displayed a pronounced signal reduction in the tumor region, as compared to the contralateral normal tissue. Whereas no apparent difference between the tumor and normal regions was found in NOE −3.5 ppm maps, the NOE −1.6 ppm map showed relatively hypointensity in the tumor region. All the amplitude maps fitted by IDEAL method appeared more homogeneous than those from conventional fitting. We also compared the CEST contrasts between the tumor and contralateral normal regions in all the animals (N = 8). Both methods showed a significantly higher APT signal (P < 0.001, paired t-test) and a strong negative MT contrast (P < 0.001, paired t-test) in the tumor. No significant change in NOE −3.5 ppm signal was found in the tumor (P = 0.16 and 0.69 for conventional and IDEAL fitting respectively, paired t-test). Interestingly, the contrast between tumor and normal regions at 2 ppm was significant using IDEAL fitting (P < 0.05, paired t-test) but not with conventional fitting (P = 0.20, paired t-test). Similarly, the IDEAL fitting revealed significant negative contrasts in the tumor in the NOE −1.6 ppm map (P < 0.01, paired t-test) which was not observed in the map from conventional fitting (P = 0.12, paired t-test). The fitted offset and linewidth maps were generally homogenous throughout the brain for both methods, but the results from conventional fitting were noisier (Fig. 6b,c).

**Figure 7:**
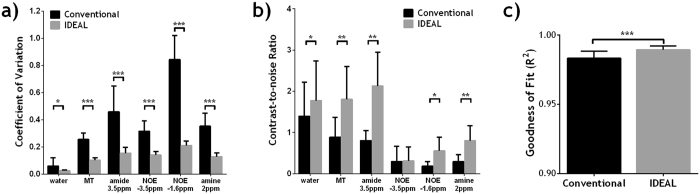
Comparison of fitting quality of conventional fitting and IDEAL fitting in rat gliomas (N = 8), including (**a**) the coefficient of variation (COV, i.e., S.D./mean) of the whole brain, (**b**) contrast-to-noise ratio (CNR) between the normal brain and brain tumor, (**c**) R^2^ of the whole brain. Paired Student's t-test was performed between the two methods with *P < 0.05, **P < 0.01 and ***P < 0.001.

Figure 7 compares the quality of IDEAL and conventional fitting methods *in vivo* in rat gliomas. The whole-brain COV in the fitted amplitude maps was significantly smaller using IDEAL fitting for all exchangeable pools (Fig. 7a). More importantly, IDEAL fitting provides substantially higher CNR in five out of the six pools (Fig. 7b). For all the gliomas studied (N = 8), the goodness of fit (R^2^) of the whole brain was significantly higher using IDEAL fitting (P < 0.001, paired t-test).

## Discussion

In this study, we proposed a new fitting algorithm that harnesses the high SNR of downsampled images for iterative fitting and achieved reliable CEST MRI quantification. This is important because *in vivo* CEST effect is subtle, typically a few percent, with concomitant RF saturation effects that are often non-negligible. The SNR of CEST images can be typically improved by increasing the number of signal averages. However, this prolongs scan time, especially for Z-spectral acquisition. Alternatively, post-processing methods such as spatial smoothing and/or spectral interpolation have been used to achieve higher SNR before multi-pool Lorentzian fitting on a voxel basis^34, 35^. Our proposed IDEAL fitting approach combined spline interpolation and spatial downsampling to achieve higher SNR for iterative fitting. On the other hand, proper selection of initial values is critical for *in vivo* CEST fitting, particularly for cases of complex multi-component systems under suboptimal SNR. The proposed IDEAL fitting approach avoids arbitrary selection of initial values and minimizes operator bias, enabling automated and adaptive fitting for reliable estimation of CEST effects. We first evaluated the performance of IDEAL fitting in a CEST phantom. Overall, the IDEAL fitting results are of smaller COV, higher CNR and comparable goodness of fit at a faster fitting speed when compared to those of the conventional fitting. The fitted maps from the IDEAL method were generally less noisy, even with the raw CEST images plagued by superimposed noise, likely due to the good initial values determined from high SNR Z-spectra. The use of tightly constrained boundaries after initial fitting can minimize undesirable fluctuations in fitting results due to superimposed noise or poor SNR.

We further applied the IDEAL fitting to quantify the contributions from APT, amine, NOE and MT effects in a rat model of glioma. A substantially higher APT signal in the tumor was in line with the previous findings using APT imaging of glioma^28, 36–38^, likely attributable to elevated intracellular mobile proteins/peptides concentration^39^. Both methods showed a strong negative MT contrast in the tumor, consistent with the finding from quantitative MT^40^. In addition, no significant changes in NOE-3.5 ppm were found in the tumor, similar to the previous studies using similar irradiation powers^28, 41, 42^. Interestingly, the IDEAL fitting revealed a pronounced negative contrast in the tumor in the fitted amine (2 ppm) map which was not observed in the map from conventional fitting. The CEST effect at 2 ppm has been shown to correlate with the creatine level in brain tumor and change with tumor progression^4, 43^. We also found a significantly reduction in NOE-1.6 ppm, which was also observed in a recent CEST study of rat glioma at 9.4 T^44^. It has been suggested that this effect may originate from choline phospholipids and can be used as a new biomarker for the detection of brain tumor. Indeed, the CNR between the tumor and normal region in the amine and NOE-1.6 ppm map was much smaller than 1 using conventional method. Therefore, the IDEAL fitting is advantageous in revealing such changes under suboptimal SNR.

Although it has been shown that both labile proton concentration and exchange rate can be simultaneously determined from quantitative CEST (qCEST) analysis, its *in vivo* application is somewhat cumbersome due to the presence of complex multiple exchangeable compartments. It is worthwhile to point out that Lorentzian decoupling provides reasonable CEST quantification, particularly under the case of relatively weak RF saturation where different CEST peaks can be reliably detected (Fig. S2). The use of strong saturation levels may enhance the CEST effects but it also leads to increased RF spillover and broadened peaks that may substantially overlap with one another, resulting in less reliable decoupling results. Because the typical exchange rates from amide protons, MT and NOE components for *in vivo* CEST MRI are relatively slow, and therefore, it is advantageous to choose a relatively weak RF irradiation level for both experimental optimization and quantification. Moreover, the proposed IDEAL method takes advantage of the high SNR of global Z-spectrum so the peaks of CEST effects can be reliably identified. The proposed IDEAL approach may be further combined with qCEST analysis. Decoupling the complex CEST contrast is highly desired for more accurate quantification using qCEST analysis such as ratiometric^45^ or omega plot approaches^46, 47^, which will be investigated in our future work. In this study, routine T_1_ and T_2_ maps were acquired to identify the tumors (Fig. S1, supplementary information). Indeed, significantly prolonged T_1_ and reduced T_2_ were found in the tumor region (Table S1). In the future, it is worthwhile to evaluate the correlation between relaxation times and decoupled CEST contrasts in gliomas, which may assist in the optimization of quantitative CEST for cancer imaging. Additionally, we would like to point out the limitation of using R^2^ to assess the goodness of fit, which may not faithfully reflect the fitting quality, especially with a multi-pool model at suboptimal SNR. In the case of conventional fitting, relatively high R^2^ can be achieved through relaxed boundary constraints but the fitting results may substantially deviate from those determined under excellent SNR. In addition, the loose boundary constraints may likely lead to longer computing time as more iterative steps were taken to solve the ‘best’ fit. One limitation of the IDEAL method is that image downsampling will introduce aliasing while upscaling the fitted maps may lead to reduced contrast and produce staircase artifacts around edges. It is necessary to choose anti-aliasing algorithm and edge-directed interpolation to preserve the edges after scaling.

Recently, multi-pool Lorentzian fitting has been increasingly employed to isolate the APT and NOE effect from the spillover and MT confounded Z-spectrum^27–29, 32, 33^. However, such post-processing routines either require acquisition of Z-spectra of wide range of frequency offsets or are limited to cases of prominent APT/NOE effects. On the other hand, the SNR of CEST Z-spectral images can be compromised due to high spatialtemporal resolution and/or the need to obtain densly-sampled Z-spectra quickly. As such, the SNR may be suboptimal for conventional fitting routine. Under such conditions, the proposed IDEAL fitting facilitates CEST quantification, quickly and reliably. Moreover, the IDEAL fitting can be applied to noise prone MRI dataset with a fourth dimension, such as blood oxygenation level dependent (BOLD) functional MRI, dynamic contrast-enhanced (DCE) MRI, and dynamic susceptibility contrast (DSC) MRI, etc.

In summary, the proposed IDEAL fitting method quantifies CEST images based on initial values from multi-pool Lorentzian fitting of iteratively less downsampled images until quantitative CEST maps of desired resolution are obtained. It allows reliable fitting at a faster fitting speed, and is less susceptible to suboptimal SNR than the conventional method. The CEST maps fitted using the IDEAL method showed smaller COV and higher CNR, which are desirable for *in vivo* CEST quantification. The IDEAL fitting was applied to quantify CEST effects in rat gliomas and the results showed significant changes in APT and MT effects in tumors similar to those of conventional method but with substantially higher CNRs. More importantly, it revealed pronounced negative contrasts in tumors in the fitted CEST maps at 2 ppm and −1.6 ppm which were not found using conventional method due to strong variation in its fitting results. These CEST contrasts may reflect changes in creatine level and NOE effects, warranting further investigation of their potentials in tumor detection, grading and treatment. It is anticipated that the proposed IDEAL fitting can be generalized to quantify MRI dataset where SNR is suboptimal.

## Materials and Methods

### Phantom

We prepared an *in vitro* CEST phantom with two vials containing different concentrations of mixed creatine and nicotinamide in CuSO_4_-doped (1.5 mM) phosphate buffered solution (Sigma Aldrich, St Louis, MO). The concentration of creatine in the two vials was 50 mM and 100 mM and that of nicotinamide was 100 mM and 50 mM; pH was titrated to 7.0 (EuTech Instrument, Singapore). The vials were then sealed and inserted into a 50 ml falcon tube filled with 1.5% low gelling-point agarose.

### Tumor Model

Animal experiments were performed in accordance with institutional guidelines, as approved by the Institutional Animal Care and Use Committees (IACUC), Massachusetts General Hospital. 2 × 10^5^ cells of the non-infiltrating D74-rat glioma model^48, 49^ were injected into the right frontal lobe of adult male Fischer 344 rats (N = 8), as previously described^50^. The animals were imaged 11–13 days after tumor implantation.

### MRI

MRI scans were performed on a 4.7 Tesla small-bore scanner (Bruker Biospec, Ettlingen, Germany). For the phantom study, CEST MRI was obtained with single-slice, single-shot echo planar imaging (EPI) (field of view (FOV) = 52 × 52 mm^2^, matrix = 96 × 96, slice thickness = 10 mm). Z-spectrum was acquired from −5 ppm to 5 ppm with intervals of 0.125 ppm and RF irradiation power level of 1 µT. The repetition time (TR)/saturation time (TS)/echo time (TE) was 10 s/5 s/45 ms, number of average (NSA) = 1, and the total scan time was 13 min 40 s. For the rodent study, multi-slice MRI (FOV = 20 × 20 mm^2^, matrix = 64 × 64, 5 slices, slice thickness/gap = 1.8/0.2 mm) was acquired with single-shot EPI. We obtained Z-spectrum from −6 ppm to 6 ppm with intervals of 0.25 ppm and RF irradiation power level of 0.75 µT, TR/TS/TE = 10 s/5 s/15 ms, NSA = 2 and scan time = 8 min 20 s. In addition, T_1_-weighted images were acquired with seven inversion delays ranging from 250 ms to 3,000 ms (TR/TE = 6,500/28 ms, NSA = 4); T_2_-weigthed images were obtained with two TE of 30 and 100 ms (TR = 3,250 ms, NSA = 16)^51^.

### Data Analysis

Data were processed in MATLAB (MathWorks, Natick, MA). T_1w_ and T_2w_ parametric maps were obtained with least-squares mono-exponential fitting of the signal intensities as a function of inversion time and TE, respectively. Z-spectra (I) were interpolated by smoothing splines, corrected for field inhomogeneity using a WASSR map and normalized by the signal without RF irradiation (I_0_)^52, 53^. For the conventional fitting method, multi-pool Lorentzian fitting of the Z-spectra on a voxel basis was applied to estimate the CEST effects from different pools^4, 24^. Briefly, the Z-spectrum was fitted as the sum of multiple Lorentzian functions with the following equation

1 $$1-\frac{I}{{I}_{0}}=\sum _{i=1}^{N}\frac{{A}_{i}}{1+4{(\frac{\omega -{\omega }_{i}}{{\sigma }_{i}})}^{2}}$$

where *ω* is the frequency offset from the water resonance, *A*_*i*_, *ω*_*i*_ and *σ*_*i*_ are the amplitude, frequency offset and linewidth of the CEST peak for the i^th^ proton pool, respectively. In the CEST phantom, we employed a five-pool Lorentzian model, including water (0 ppm), magnetization transfer (MT, −2 ppm), amide (3.5 ppm), creatine (1.9 ppm) and hydroxyl (-OH, 1 ppm). Note the frequency offset of each pool was used as initial values for fitting. For the *in vivo* study of rat gliomas, a six-pool Lorentzian model of water (0 ppm), MT (−2 ppm), amide (3.5 ppm), amine (2 ppm) and Nuclear Overhauser enhancement (NOE) effects at 3.5 ppm and 1.6 ppm upfield from water was used^44, 54^.

The conventional fitting was constrained by bounds between 10% and 10 times of the initial values for amplitude and linewidth, and ±20% of the corresponding linewidth for frequency offset of each proton pool. Figure 1 shows the flow chart of IDEAL fitting. Briefly, a global Z-spectrum was first obtained by averaging the Z-spectra from all voxels within the mask. Multi-pool Lorentzian fitting of the global Z-spectrum with the same loosely constrained bounds as the conventional fitting to determine initial values for subsequent fine fitting. The field inhomogeneity-corrected CEST images were downsampled to 4 × 4 for voxel-wise multi-pool Lorentzian fitting, with the initial values of each voxel determined by the fitted values of the global Z-spectrum and tightly constrained bounds of ±10% the initial values for amplitude and linewidth, and ±5% of the corresponding linewidth for frequency offset. The same procedure was repeated sequentially for the CEST images downsampled to 8 × 8, 16 × 16, 32 × 32, etc., till the original image size of 96 × 96 or 64 × 64, with the initial values of each voxel determined by interpolating the fitted amplitude/linewidth/offset maps of the last downsampled images. For both fitting methods, the quality of fitting was evaluated by (1) coefficient of variation (COV, i.e., standard deviation/mean) within the mask in the fitted amplitude maps, (2) contrast-to-noise ratio (CNR) in the fitted amplitude maps between the two vials in the phantom or between the normal and tumor regions calculated by $CNR=\frac{| {S}_{1}-{S}_{2}| }{\sqrt{({\sigma }_{1}^{2}+{\sigma }_{2}^{2})}}$, where *S*_*1,2*_ are the mean values for the two regions-of-interest (ROIs) respectively and *σ*_*1,2*_ are their standard deviations, and (3) goodness of fit (R^2^) maps. The ROIs of tumor tissues were defined based on the distinct information from T_1w_ and T_2w_ maps and mirrored to the contralateral hemisphere for the ROIs of normal tissues (Fig. S1). To evaluate the performance of the fitting methods under noise, different levels of Gaussian noise with σ ranging from 0.005 to 0.1, were added to the original phantom data to test the performance of both fitting algorithms.

## Electronic supplementary material

Supplementary Information

## References

[1] Sherry AD, Woods M (2008). Chemical exchange saturation transfer contrast agents for magnetic resonance imaging. Annual review of biomedical engineering.

[2] van Zijl PC, Yadav NN (2011). Chemical exchange saturation transfer (CEST): what is in a name and what isn’t?. Magnetic resonance in medicine.

[3] Ward KM, Aletras AH, Balaban RS (2000). A new class of contrast agents for MRI based on proton chemical exchange dependent saturation transfer (CEST). Journal of magnetic resonance.

[4] Cai K (2015). CEST signal at 2 ppm (CEST@2 ppm) from Z-spectral fitting correlates with creatine distribution in brain tumor. NMR in biomedicine.

[5] Walker-Samuel S (2013). *In vivo* imaging of glucose uptake and metabolism in tumors. Nature medicine.

[6] Chan KW (2012). Natural D-glucose as a biodegradable MRI contrast agent for detecting cancer. Magnetic resonance in medicine.

[7] Cai K (2012). Magnetic resonance imaging of glutamate. Nature medicine.

[8] Zhang S, Malloy CR, Sherry AD (2005). MRI thermometry based on PARACEST agents. Journal of the American Chemical Society.

[9] Zhou J, Payen JF, Wilson DA, Traystman RJ, van Zijl PC (2003). Using the amide proton signals of intracellular proteins and peptides to detect pH effects in MRI. Nature medicine.

[10] Sun PZ, Wang E, Cheung JS (2012). Imaging acute ischemic tissue acidosis with pH-sensitive endogenous amide proton transfer (APT) MRI–correction of tissue relaxation and concomitant RF irradiation effects toward mapping quantitative cerebral tissue pH. NeuroImage.

[11] Guo Y (2016). pH-sensitive MRI demarcates graded tissue acidification during acute stroke - pH specificity enhancement with magnetization transfer and relaxation-normalized amide proton transfer (APT) MRI. NeuroImage.

[12] Sun PZ, Zhou J, Sun W, Huang J, van Zijl PC (2007). Detection of the ischemic penumbra using pH-weighted MRI. Journal of cerebral blood flow and metabolism: official journal of the International Society of Cerebral Blood Flow and Metabolism.

[13] Tietze A (2014). Assessment of ischemic penumbra in patients with hyperacute stroke using amide proton transfer (APT) chemical exchange saturation transfer (CEST) MRI. NMR in biomedicine.

[14] Sun PZ, Cheung JS, Wang E, Lo EH (2011). Association between pH-weighted endogenous amide proton chemical exchange saturation transfer MRI and tissue lactic acidosis during acute ischemic stroke. Journal of cerebral blood flow and metabolism: official journal of the International Society of Cerebral Blood Flow and Metabolism.

[15] Zaiss M (2014). Inverse Z-spectrum analysis for spillover-, MT-, and T1 -corrected steady-state pulsed CEST-MRI–application to pH-weighted MRI of acute stroke. NMR in biomedicine.

[16] Jia G (2011). Amide proton transfer MR imaging of prostate cancer: A preliminary study. J Magn Reson Imaging.

[17] Xu J (2014). On the origins of chemical exchange saturation transfer (CEST) contrast in tumors at 9.4 T. NMR in biomedicine.

[18] Desmond KL, Moosvi F, Stanisz GJ (2014). Mapping of amide, amine, and aliphatic peaks in the CEST spectra of murine xenografts at 7 T. Magnetic resonance in medicine.

[19] Chen LQ (2014). Evaluations of extracellular pH within *in vivo* tumors using acidoCEST MRI. Magnetic resonance in medicine.

[20] Sagiyama K (2014). *In vivo* chemical exchange saturation transfer imaging allows early detection of a therapeutic response in glioblastoma. Proc Natl Acad Sci USA.

[21] Haris M (2014). *In vivo* Magnetic Resonance Imaging of Tumor Protease Activity. Sci Rep.

[22] Jin T, Wang P, Zong X, Kim SG (2013). MR imaging of the amide-proton transfer effect and the pH-insensitive nuclear overhauser effect at 9.4 T. Magnetic resonance in medicine.

[23] Sun PZ, Benner T, Copen WA, Sorensen AG (2010). Early experience of translating pH-weighted MRI to image human subjects at 3 Tesla. Stroke.

[24] Zaiss M (2015). Relaxation-compensated CEST-MRI of the human brain at 7T: Unbiased insight into NOE and amide signal changes in human glioblastoma. NeuroImage.

[25] Windschuh J (2015). Correction of B1-inhomogeneities for relaxation-compensated CEST imaging at 7 T. NMR in biomedicine.

[26] Yoo B (2014). Detection of *in vivo* enzyme activity with CatalyCEST MRI. Magnetic resonance in medicine: official journal of the Society of Magnetic Resonance in Medicine/Society of Magnetic Resonance in Medicine.

[27] Heo HY (2015). Whole-brain amide proton transfer (APT) and nuclear overhauser enhancement (NOE) imaging in glioma patients using low-power steady-state pulsed chemical exchange saturation transfer (CEST) imaging at 7T. J Magn Reson Imaging.

[28] Heo HY, Zhang Y, Lee DH, Hong X, Zhou J (2016). Quantitative assessment of amide proton transfer (APT) and nuclear overhauser enhancement (NOE) imaging with extrapolated semi-solid magnetization transfer reference (EMR) signals: Application to a rat glioma model at 4.7 Tesla. Magnetic resonance in medicine.

[29] Zaiss M, Schmitt B, Bachert P (2011). Quantitative separation of CEST effect from magnetization transfer and spillover effects by Lorentzian-line-fit analysis of z-spectra. Journal of magnetic resonance.

[30] Zaiss M, Bachert P (2013). Chemical exchange saturation transfer (CEST) and MR Z-spectroscopy *in vivo*: a review of theoretical approaches and methods. Physics in medicine and biology.

[31] Liu G, Song X, Chan KW, McMahon MT (2013). Nuts and bolts of chemical exchange saturation transfer MRI. NMR in biomedicine.

[32] Jones CK (2013). Nuclear Overhauser enhancement (NOE) imaging in the human brain at 7T. NeuroImage.

[33] Desmond KL, Moosvi F, Stanisz GJ (2014). Mapping of amide, amine, and aliphatic peaks in the CEST spectra of murine xenografts at 7 T. Magn. Reson. Med..

[34] Moon BF (2015). A comparison of iopromide and iopamidol, two acidoCEST MRI contrast media that measure tumor extracellular pH. Contrast media & molecular imaging.

[35] Longo DL (2014). A general MRI-CEST ratiometric approach for pH imaging: demonstration of *in vivo* pH mapping with iobitridol. Journal of the American Chemical Society.

[36] Zhou J, Lal B, Wilson DA, Laterra J, van Zijl PC (2003). Amide proton transfer (APT) contrast for imaging of brain tumors. Magnetic resonance in medicine.

[37] Jones CK (2006). Amide proton transfer imaging of human brain tumors at 3T. Magnetic resonance in medicine.

[38] Salhotra A (2008). Amide proton transfer imaging of 9L gliosarcoma and human glioblastoma xenografts. NMR in biomedicine.

[39] Yan K (2015). Assessing Amide Proton Transfer (APT) MRI Contrast Origins in 9 L Gliosarcoma in the Rat Brain Using Proteomic Analysis. Molecular imaging and biology: MIB: the official publication of the Academy of Molecular Imaging.

[40] Xu J (2014). On the origins of chemical exchange saturation transfer (CEST) contrast in tumors at 9.4 T. NMR in biomedicine.

[41] Lee, D. H. *et al.* Quantitative assessment of the effects of water proton concentration and water T changes on amide proton transfer (APT) and nuclear overhauser enhancement (NOE) MRI: The origin of the APT imaging signal in brain tumor. *Magnetic resonance in medicine*, doi:10.1002/mrm.26131 (2016). 10.1002/mrm.26131 PMC4970958 26841096

[42] Scheidegger R, Wong ET, Alsop DC (2014). Contributors to contrast between glioma and brain tissue in chemical exchange saturation transfer sensitive imaging at 3 Tesla. NeuroImage.

[43] Cai, K. *et al*. Creatine CEST MRI for Differentiating Gliomas with Different Degrees of Aggressiveness. *Molecular imaging and biology: MIB: the official publication of the Academy of Molecular Imaging*, doi:10.1007/s11307-016-0995-0 (2016). 10.1007/s11307-016-0995-0 PMC5824619 27541025

[44] Zhang, X.-Y. *et al.* MR imaging of a novel NOE-mediated magnetization transfer with water in rat brain at 9.4 T. *Magnetic Resonance in Medicine*, n/a–n/a, doi:10.1002/mrm.26396 (2016). 10.1002/mrm.26396 PMC5342948 27604612

[45] Sun PZ (2012). Simplified quantification of labile proton concentration-weighted chemical exchange rate (k(ws) ) with RF saturation time dependent ratiometric analysis (QUESTRA): normalization of relaxation and RF irradiation spillover effects for improved quantitative chemical exchange saturation transfer (CEST) MRI. Magnetic resonance in medicine: official journal of the Society of Magnetic Resonance in Medicine/Society of Magnetic Resonance in Medicine.

[46] Dixon WT (2010). A concentration-independent method to measure exchange rates in PARACEST agents. Magnetic resonance in medicine: official journal of the Society of Magnetic Resonance in Medicine/Society of Magnetic Resonance in Medicine.

[47] Wu R, Xiao G, Zhou IY, Ran C, Sun PZ (2015). Quantitative chemical exchange saturation transfer (qCEST) MRI - omega plot analysis of RF-spillover-corrected inverse CEST ratio asymmetry for simultaneous determination of labile proton ratio and exchange rate. NMR in biomedicine.

[48] Barth RF, Kaur B (2009). Rat brain tumor models in experimental neuro-oncology: the C6, 9L, T9, RG2, F98, BT4C, RT-2 and CNS-1 gliomas. Journal of neuro-oncology.

[49] Barth RF (1998). Rat brain tumor models in experimental neuro-oncology: the 9L, C6, T9, F98, RG2 (D74), RT-2 and CNS-1 gliomas. Journal of neuro-oncology.

[50] Fulci G (2006). Cyclophosphamide enhances glioma virotherapy by inhibiting innate immune responses. Proc Natl Acad Sci USA.

[51] Cheung, J. S., Wang, E. F., Zhang, X. A., Manderville, E., Lo, E. H., Sorensen, A. G. & Sun, P. Z. Fast radio-frequency enforced steady state (FRESS) spin echo MRI for quantitative T2 mapping: minimizing the apparent repetition time (TR) dependence for fast T2 measurement. **NMR Biomed****25**, 189–194 (2012). 10.1002/nbm.1729 PMC3712835 21755552

[52] Stancanello J (2008). Development and validation of a smoothing-splines-based correction method for improving the analysis of CEST-MR images. Contrast media & molecular imaging.

[53] Kim M, Gillen J, Landman BA, Zhou J, van Zijl PCM (2009). Water saturation shift referencing (WASSR) for chemical exchange saturation transfer (CEST) experiments. Magnetic resonance in medicine.

[54] Zhang XY (2016). A new NOE-mediated MT signal at around -1.6 ppm for detecting ischemic stroke in rat brain. Magnetic resonance imaging.

