# Supplementary material for: Quantitative chemical exchange saturation transfer (CEST) MRI of glioma using Image Downsampling Expedited Adaptive Least-squares (IDEAL) fitting
Source: Sci Rep. 2017 Mar 7;7:84. doi: 10.1038/s41598-017-00167-y (PMC5427899; doi:10.1038/s41598-017-00167-y)
Supplement: Supplementary file 1 — Supplementary Information [file 41598_2017_167_MOESM1_ESM.doc]

**Quantitative chemical exchange saturation transfer (CEST) MRI of glioma using Image Downsampling Expedited Adaptive Least-squares (IDEAL) fitting**

Iris Yuwen Zhou ^1^‡, Enfeng Wang ^1,2^‡, Jerry S Cheung ^1^, Xiaoan Zhang ^2^, Giulia Fulci ^3^, Phillip Zhe Sun ^1^*

^1^ Athinoula A. Martinos Center for Biomedical Imaging, Department of Radiology, Massachusetts General Hospital and Harvard Medical School, Charlestown, MA, USA

^2^ Department of Radiology, 3^rd^ Affiliated Hospital, Zhengzhou University, Henan, China

^3^ Molecular Neuro-oncology Laboratories, Department of Neurosurgery, Massachusetts General Hospital and Harvard Medical School, Boston, MA 02124, USA

**Supplementary Information**


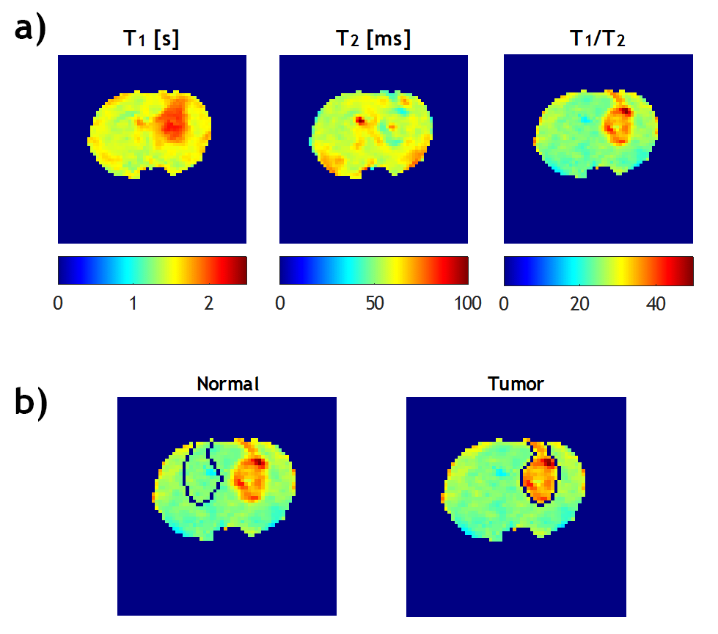


**Figure S1. a)** Relaxation maps of a representative rat with a D74 glioma tumor. In the T1 map, the tumor core shows the strongest contrast but the elevated T1 gradually reduced from the core to the tumor rim, resulting in an obscure boundary of the tumor. In contrast, substantially lower T2 can be found in the tumor rim but not in the tumor core. **b)** We took advantage of the distinct features of the relaxation maps and defined the tumor ROIs based on the T1/T2 map and mirrored to the contralateral hemisphere as the ROIs of normal tissues.

**
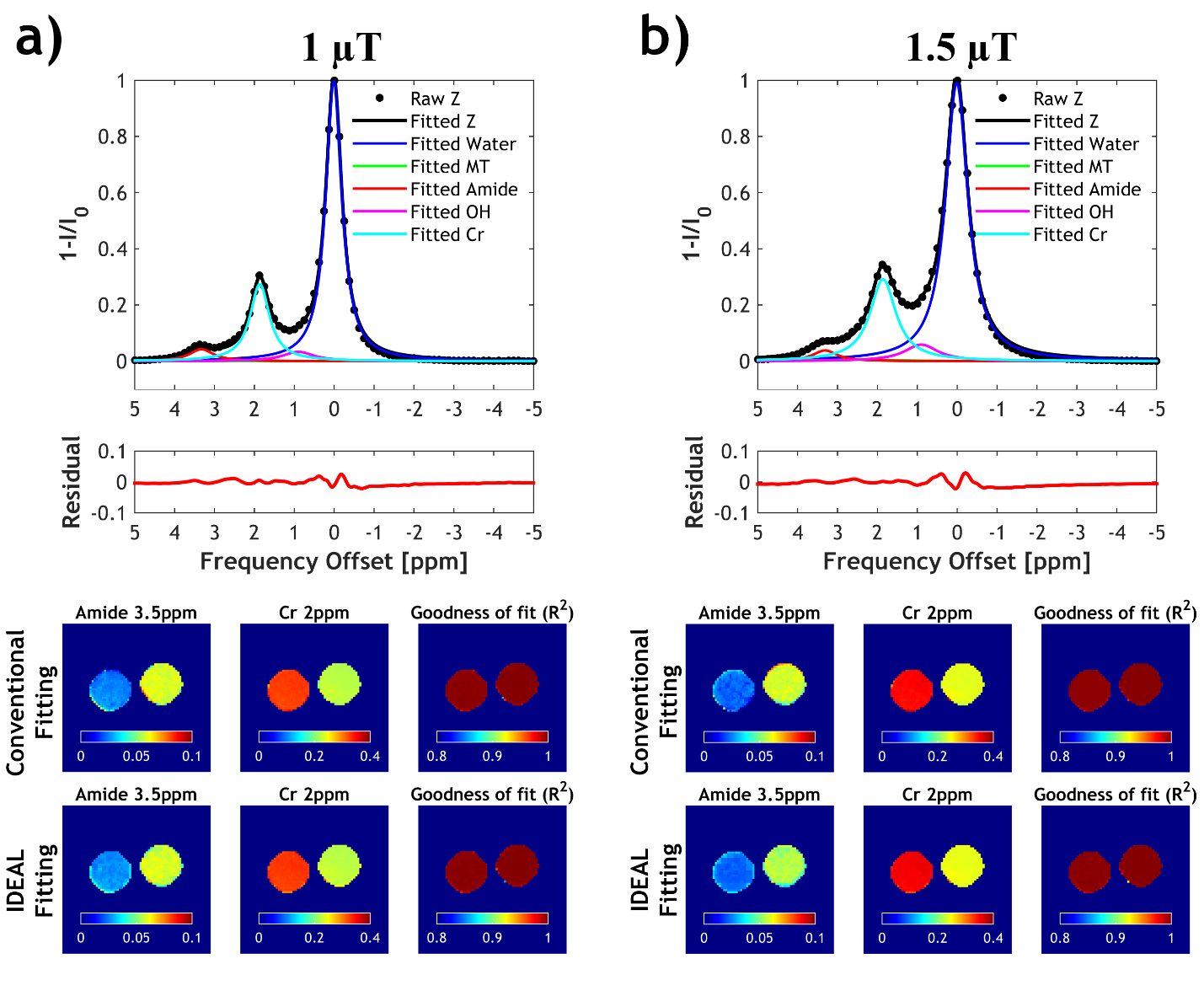
**

**Figure S2.** Multi-pool Lorentzian fit of the global Z spectra from a two-compartment CEST phantom at RF saturation power of **a)** 1 µT and **b)** 1.5 µT to determine initial values for IDEAL fitting (Top). The fitting result from conventional voxel-wise multi-pool Lorentzian fitting was compared with that from the IDEAL fitting. The CEST phantom contains left: 50 mM Creatine (Cr) and 100 mM nicotinamide (amide); right: 100 mM Cr and 50mM amide.

**Table S1** Comparison of longitudinal relaxation time (T1) and transverse relaxation time in normal and tumor area. Paired Student’s t-test was performed.

|  | **Normal** | **Tumor** | **p-value** |
| --- | --- | --- | --- |
| **T_1_ [s]** | 1.490 ± 0.077 | 1.956 ± 0.078 | <0.001 |
| **T_2_ [ms]** | 56.1 ± 2.1 | 53.4 ± 1.9 | 0.002 |
